# Supplementary material for: Association between non-acute traumatic injury (TI) and heart rate variability (HRV) in adults: A systematic review protocol
Source: PLoS One. 2022 Aug 26;17(8):e0273688. doi: 10.1371/journal.pone.0273688 (PMC9417186; doi:10.1371/journal.pone.0273688)
Supplement: S2 Table — (DOCX) [file pone.0273688.s002.docx]

Supporting Information 2 (Table): Supplementary study selection criteria

| 1. Does the exposure consist of traumatic physical injury? (e.g., burn, abdominal, thoracic, fall etc)   No: Stop. Excluded (Not relevant to topic)  Yes: Proceed to 2. |
| --- |
| 1. Is the study on human adults?   No: Stop. Excluded (Not relevant to topic)  Yes: Proceed to 3 |
| 1. Is the article of any following study designs or publication types: Case report, Case series study, Review article, Systematic Review, Opinion/Editorial, In-vitro and animal study?   No: Proceed to 4.  Yes: Stop. Excluded (Excluded study design or publication type) |
| 1. Does the article report on any of the heart rate variability outcomes (e.g., time, frequency and non-linear measure) as a primary or secondary outcome?   No: Stop. Excluded (Not relevant to topic)  Yes: Proceed to 5. |
| 1. Does the article deal with traumatic brain injury (TBI) or Post-Traumatic Stress Disorder (PTSD)?   No: Proceed to 6  Yes: Stop (Psychological trauma and traumatic brain injuries are excluded as they influence autonomic nervous system) |
| 1. Does the article deal with selected populations of other non-traumatic injury? (e.g., infection and inflammation etc)   No: Proceed.  Yes: Stop (wrong population- very high risk of bias) |
