# Supplementary material for: Association between non-acute traumatic injury (TI) and heart rate variability (HRV) in adults: A systematic review protocol
Source: PLoS One. 2022 Aug 26;17(8):e0273688. doi: 10.1371/journal.pone.0273688 (PMC9417186; doi:10.1371/journal.pone.0273688)
Supplement: Attachment — Submitted filename: reviewer report.docx [file pone.0273688.s004.docx]

I carefully studied the new file. The importance of the work in the introduction should be written thoroughly with up-to-date sources. The article is also in the discussion section Too short and too weak. It is not possible to be published in this way. It should be discussed with the articles included in the study. This section must be developed.
