# Supplementary material for: Association between non-acute traumatic injury (TI) and heart rate variability (HRV) in adults: A systematic review protocol
Source: PLoS One. 2022 Aug 26;17(8):e0273688. doi: 10.1371/journal.pone.0273688 (PMC9417186; doi:10.1371/journal.pone.0273688)
Supplement: Attachment — Submitted filename: Response to reviewers.docx [file pone.0273688.s005.docx]

PONE-D-22-12255: Association between non-acute traumatic injury (TI) and heart rate variability (HRV) in adults: a systematic review protocol

We would like to thank the academic editor and the reviewers for providing insightful feedback on our manuscript which has improved our paper. The suggestions made by the academic editor and the reviewers have been incorporated into the manuscript and are highlighted. Please see below, in blue, for a point-by-point response to the reviewers’ comments.

Academic Editor:

Dear authors,
The present study protocol is interesting but there still some improvements that can be made before further consideration. For instance, some methodological aspects need more rational and support such as, the characteristics of the target population in study, the inclusion/exclusion criteria, and the development of some preliminary results and discussion to stretch the importance of such study.
Please consider the comments made by the reviewers.

Response: Thank you for the feedback. Previously, for brevity, some methodological aspects were only briefly discussed. However, in the light of your helpful comments, the above suggestions have now been incorporated into our revised manuscript and are tracked.

Reviewer #1: The present study is of interest to investigate the association between non-acute traumatic injury (TI) and heart rate variability (HRV) in adults, throughout a systematic review protocol.

Response: Thank you!

Despite the interesting work, I strongly suggest following the comments to improve the quality of the manuscript.

1. Line 137 and 138 Population: "We aim to include studies with adult human participants aged 18 or above with no restriction on gender and occupation." Please explain why?

Response: Thanks for this comment. This sentence is worded in this way to emphasise that our review is intended to be as inclusive as possible in terms of incorporating both men and women as well as a broad spectrum of participants (e.g. civilian and military). The following changes have been made (line 141-142).

*“The rationale is to include a broad spectrum of participants (civilian and military) [3, 4, 5].”*

Please note that the line numbers originally quoted by the reviewers have changed due to some addition in the revised manuscript.

2. Line 139-140: "Studies with participants who sustained chronic TIs (>7 days post injury) and post initial hospital discharge will be included instead of acute injuries which led to death upon hospital admission." Please explain why?

Response: Apologies for the confusion. We agree that this needed more explanation. Setting the background, the following lines have now been added in the introduction section:

(Line 84-85): *In the last two decades, research into the impact of TI on HRV has been largely focused on acute trauma and during hospital admission [14, 15, 16, 17].*

(Line 88-90): *(the current knowledge gap is in non-acute TI and HRV with the need to include multiple injury types. To date, no such systematic review has been undertaken)”.*

-and under the heading “Eligibility criteria”:

(Line 145-147): *We selected the cut-off point of >7 days post injury based on a previous review on mild TBI [29] and to mitigate the impact of early physiological impact of acute trauma on HRV [18, 19, 20, 21].*

In sum, the rationale is that previous systematic reviews have looked at the relationship between HRV and acute trauma (selected TIs such as spinal cord, traumatic brain injury). To address this research gap, our systematic review will examine the association between non-acute (or generalised) TI and HRV in adults.

Seven new references have been added (see below). Therefore, the in-text citations and reference list have been updated (tracked).

- Batchinsky AI, Wolf SE, Molter N, Kuusela T, Jones JA, Moraru C, Boehme M, Williams K, Bielke P, Wade C, Holcomb JB, Cancio LC. Assessment of cardiovascular regulation after burns by nonlinear analysis of the electrocardiogram. J Burn Care Res*.* 2008;29(1):56-63.
- Colombo J, Shoemaker WC, Belzberg H, Hatzakis G, Fathizadeh P, Demetriades D. Noninvasive monitoring of the autonomic nervous system and hemodynamics of patients with blunt and penetrating trauma. J Trauma Acute Care Surg. 2008;65(6):1364-73.
- Riordan Jr WP, Norris PR, Jenkins JM, Morris Jr JA. Early loss of heart rate complexity predicts mortality regardless of mechanism, anatomic location, or severity of injury in 2178 trauma patients. J Surg Res*.* 2009;156(2):283-9.
- Mowery NT, Morris Jr JA, Jenkins JM, Ozdas A, Norris P. Core temperature variation is associated with heart rate variability independent of cardiac index: a study of 278 trauma patients. J Crit Care 2011;26(5):534-e9.
- Buker DB, Oyarce CC, Plaza RS. Effects of spinal cord injury in heart rate variability after acute and chronic exercise: a systematic review. Top Spinal Cord Inj. 2018;24(2):167-76.
- Krainin BM, Forsten RD, Kotwal RS, Lutz RH, Guskiewicz KM. Mild traumatic brain injury literature review and proposed changes to classification. J Spec Oper Med. 2011;11(3):38-47.
- Bhatnagar V, Richard E, Melcer T, Walker J, Galarneau M. Retrospective study of cardiovascular disease risk factors among a cohort of combat veterans with lower limb amputation. Vasc Health Risk Manag. 2019;15:409.

3. Table1. Authors should explain why this inclusion/exclusion criteria were chosen across the text. Please use valid references to support your rationale.

Response: Thank you for raising this. In our introduction, we have presented the background and rational of our target population as well as highlighting some of the key research gaps with reference to our targeted population with the inclusion of up-to-date references. These references have now been cited in line 165-166:

*The inclusion and exclusion criteria are based on previous studies on the topic [3, 4, 18-25]*

4. Why authors decided to use RoB for the risk of bias analyses? Please clarify.

Response: Thank you for highlighting this. We decided to use RoBANS for assessing RoB due to its validity and suitability for non-randomised and observational studies. The following lines (215-220) have now been added to the manuscript to clarify this point:

*Since non-randomized studies will be included in the review, the Risk of Bias (RoB) will be assessed using the six-item Risk of Bias Assessment tool for of Non-randomized Studies (RoBANS) [33]. The following six domains will be assessed in the included studies: the selection of participants, confounding variables, the measurement of exposure, the blinding of the outcome assessments, incomplete outcome data, and selective outcome reporting [33].*

5. Despite being this a "systematic review protocol" I think that will be very interesting to present some "preliminary results" accompanied with some "preliminary discussion" (more robust) around the topic here presented. As it stands it is not clear how your questions/hypothesis will be really answered.

Response: Thanks for this suggestion. Our preliminary searches indicate a rather small body of evidence on the topic. Sharing the preliminary results may be premature at the moment. However, the anticipated limitations of the included studies (e.g. heterogeneity in terms of HRV acquisition and analysis) have been discussed in “discussion”. Similarly, the anticipated data analysis approaches have been described under “data synthesis”. Furthermore, the importance of our work in the light of current research has also been discussed (please see our response to Reviewer 2’s 2^nd^ comment).

Reviewer #2: I carefully studied the file. The importance of the work in the introduction should be written thoroughly with up-to-date sources. Also, it should be mentioned relevant studies in the introduction.

Response: Thanks for this suggestion. In the Introduction, the existing literature has been reviewed briefly from old to new studies. Addressing Reviewer 1’s comment as well, a few more studies have also been added to clarify the inclusion and exclusion criteria (Introduction section: line 84-90). References have now been cited in lines 99, 142, 146-147, 153, 166, 264). To our knowledge, all sources are up-to-date.

The article is also in the discussion section Too short and too weak. It is not possible to be published in this way. It should be discussed with the articles included in the study. This section must be developed.

Response: Thank you for highlighting this. Considering Reviewer 1’s comment 5 as well, the discussion section has now been rewritten to include the importance, strengths, and limitation of the work in the light of current research as follows (Lines 258-295):

*Traumatic injury is one of the leading causes of death in adults aged <40 years [1]. The reported adverse health consequences of injuries sustained during recent conflicts in Afghanistan and Iraq have brought the impact of TI into greater clinical focus [26].*

*The results of several recent original publications have highlighted a plausible associated between combat-related TI injury and subclinical cardiovascular risk [26, 32, 41]. The majority of this research has focussed on more well-established markers of cardiovascular risk such as heart rate, obesity, blood pressure, glucose and lipids [26]. The examination of the impact of TI on HRV has the potential to bridge the research gap. Heart rate variability is a unique non-invasive marker of autonomic balance that has been strongly linked to adverse health outcomes including MACE [17] across a broad spectrum of patient populations [14, 15, 16]. While the effects of acute TI on HRV have been studied [18, 19, 20, 21], there appears to be a paucity of research on the longer terms impacts of non-acute TI and HRV, in particular beyond that of mild traumatic brain injury [24] and spinal cord injury [25]. Examination of the longer-term effects of TI on HRV has the potential to enhance existing research knowledge gaps and offer mechanistic insight to the reported elevation of cardiovascular risk with TI.*

*Addressing this research gap, this paper presents the protocol for the systematic review of literature on the association between non-acute TIs and HRV. This systematic review is timely considering the recent trend of examining the long-term impact of TIs in vulnerable groups such as military veterans.*

*This systematic review protocol offers several strengths. Firstly, it has been registered in the PROSPERO database to ensure a transparent conduct of the systematic review. Secondly, the literature searches conducted in the 4 databases will allow a comprehensive search. Thirdly, the rigour and the quality of the included studies will be assessed using validated critical appraisal and risk of bias tools along with GRADE, independently by two reviewers. Lastly, to our knowledge, this will be the protocol of the first systematic review which examines the association between a diverse spectrum of non-acute TIs and HRV in adults. Upon completion, the systematic review will be submitted to a peer-reviewed journal. However, some limitations are also anticipated such as heterogeneity in HRV data acquisition and analysis across the studies as found in the preliminary searches.*

*Nevertheless, the importance of this systematic review is not lessened by these limitations given the transparent conduct and rigorous evaluation of the included studies using validated tools. The outcomes of this systematic review may have implications to inform trauma care practice and intervention development for civilians and vulnerable populations such as military personnel and law enforcement officers.*

Journal Requirements:

When submitting your revision, we need you to address these additional requirements.

1. Please ensure that your manuscript meets PLOS ONE's style requirements, including those for file naming. The PLOS ONE style templates can be found at
   <https://journals.plos.org/plosone/s/file?id=wjVg/PLOSOne_formatting_sample_main_body.pdf> and 
   <https://journals.plos.org/plosone/s/file?id=ba62/PLOSOne_formatting_sample_title_authors_affiliations.pdf>

Response: Thank you for this comment. To best of our knowledge, the manuscript has been written and revised according to the PLoS one’s style requirements including those for file naming. Please see revised lines 432-434. We have also followed the Study Protocol template available at PloS One website:

<https://storage.googleapis.com/plos-published-prod/c9fb/Study%20Protocol%20Article%20Template.pdf?X-Goog-Algorithm=GOOG4-RSA-SHA256&X-Goog-Credential=wombat-sa%40plos-prod.iam.gserviceaccount.com%2F20220615%2Fauto%2Fstorage%2Fgoog4_request&X-Goog-Date=20220615T101150Z&X-Goog-Expires=86400&X-Goog-SignedHeaders=host&X-Goog-Signature=1595c15be8ad346ebb2acbbb7688027e964e5dfcbb1f9663e245cdf425274c1fb484ea42eaef90dbe485c404f2643171bdfbaba483ade73457f8a32572567a0295bfc08be2a66590e6c3948e784138325fd232691b715db41f92f8bb7c80824e87a6e6fa75cfffcf6f8a904cda340cc1f0c83c4d4692fdad6266c41f1ef627e8795070419f341cf876874d35a91596e55a06fcbf90be420749757e434227a770dfdae4df567721362e6f0464b89e6b4de08810a34e34b38333440e4750d5ee79c98b4ad6d03963f56f85864bcc05310e0dcf6b2e0403217ecb2bc8221ee02211da4ba8a8e360aa8243460fcb1bec1cbbd335a31dbbc18463f0e38e7de290cdf6>

2. Thank you for stating the following in the Acknowledgments Section of your manuscript:

"We would like to acknowledge Bournemouth University and the ADVANCE charity, UK for jointly funding the PhD studentship of RM at Bournemouth University"

We note that you have provided funding information that is not currently declared in your Funding Statement. However, funding information should not appear in the Acknowledgments section or other areas of your manuscript. We will only publish funding information present in the Funding Statement section of the online submission form. 
Please remove any funding-related text from the manuscript and let us know how you would like to update your Funding Statement. Currently, your Funding Statement reads as follows:

"The authors received no specific funding for this work"

Please include your amended statements within your cover letter; we will change the online submission form on your behalf.

Response: Thank you for highlighting this. Please change the funding statement (line 32-33) to the following on my behalf:

*This project is a part of RM’s Ph.D. studentship- jointly funded by Bournemouth University and the ADVANCE charity, UK.*

The Acknowledgment statement has now been revised (line 303-304)

*We would like to acknowledge Mr. Caspian Dugdale, Academic Liaison Librarian at Bournemouth University, for helping us with building the search strategy.*
