# Supplementary material for: Association between non-acute traumatic injury (TI) and heart rate variability (HRV) in adults: A systematic review protocol
Source: PLoS One. 2022 Aug 26;17(8):e0273688. doi: 10.1371/journal.pone.0273688 (PMC9417186; doi:10.1371/journal.pone.0273688)
Supplement: Attachment — Submitted filename: Response to Reviewers.docx [file pone.0273688.s006.docx]

**PONE-D-22-12255R1:** **Association between non-acute traumatic injury (TI) and heart rate variability (HRV) in adults: a systematic review protocol**

We would like to thank the academic editor and the reviewers for providing insightful feedback on our manuscript once again. The suggestions made by the academic editor and the reviewers have been incorporated into the manuscript and are highlighted. Please see below, in blue, for a point-by-point response to the reviewers’ comments.

**Journal Requirements:**

Please review your reference list to ensure that it is complete and correct. If you have cited papers that have been retracted, please include the rationale for doing so in the manuscript text, or remove these references and replace them with relevant current references.

Response: Thank you for this comment. The reference list has now been proofread and revised according to the format available under PloS One’s reference section: <https://www.nlm.nih.gov/bsd/uniform_requirements.html>.

Any changes to the reference list should be mentioned in the rebuttal letter that accompanies your revised manuscript.

Response: Thank you for highlighting this. Below are the reference numbers 27 and 29 which have now been updated in the manuscript for the PRISMA-P checklist 2015, and 2020 guidelines, respectively:

27. Moher D, Shamseer L, Clarke M, Ghersi D, Liberati A, Petticrew M, et al. Preferred reporting items for systematic review and meta-analysis protocols (PRISMA-P) 2015 statement. Syst Rev*.* 2015;4(1):1-9.

29. Page MJ, McKenzie JE, Bossuyt PM, Boutron I, Hoffmann TC, Mulrow CD, et al. The PRISMA 2020 statement: an updated guideline for reporting systematic reviews. Syst rev. 2021;10(1):1-1.

Owing to the addition of one new reference (29), the reference list has been updated accordingly (please see the tracked changes). Following are the updated reference numbers for which a few changes have been made in the reference list related to et al. and formatting:

- 1, 2, 6,11, 13, 18, 26, 27, 29, 31, 34, 35, 37, 40, 41

Please note a few *changes* made in the manuscript as a result of proofreading:

- Line 69- ….which *measures* the fluctuations….
- Line 70-71: Deletion of abbreviations *SNS, PNS*
- Line 91-92- ..long-term *physical and psychological* impact…
- Line 110: addition of word ‘*protocol*’.
- Line 116-117: *The results will be reported in the subsequent systematic review following the PRISMA 2020 guidelines [29].*
- Line 132: Deletion of abbreviation *SCI*
- Line 151: Table 1: Deletion of repeated words *history of*
- Line 425-426: S1 Table. This is the S1 table *PRISMA-P (Preferred Reporting Items for Systematic review and Meta-Analysis Protocols) 2015 checklist.*

If you need to cite a retracted article, indicate the article’s retracted status in the References list and also include a citation and full reference for the retraction notice.

Response: Thanks for this suggestion. I can confirm that a retracted article was not cited and was removed from the reference list. We do not aim to cite any retracted article. The manuscript attached contains the complete and revised reference list.

**Additional Editor Comments:**

Dear authors,

The authors have addressed all comments of both reviewers. Nonetheless, reviewer 1 suggests that an explanation of the procedures to collect HRV should be added.

Response: Thank you. Please see our response to reviewer 1’s comment.

In addition, I found out that some methods need an update according to the most recent PRISMA 2020 guidelines, namely:
-the reference of the PRISMA 2020 update should be used;

Response: Thank you for drawing our attention to this. However, we would like highlight that the PRISMA 2020 checklist refers to the reporting of a systematic review whereas the PRISMA-P 2015 checklist is used to report a systematic review protocol- as also suggested in the recent PRISMA 2020 guidelines:

*Furthermore, PRISMA 2020 is not intended to inform the reporting of systematic review protocols, for which a separate statement is available (PRISMA for Protocols (PRISMA-P) 2015 statement* [*47*](https://www.bmj.com/content/372/bmj.n71#ref-47)*,*[*48*](https://www.bmj.com/content/372/bmj.n71#ref-48)*). (Page et al. 2021).*

Page MJ, McKenzie JE, Bossuyt PM, Boutron I, Hoffmann TC, Mulrow CD, et al. The PRISMA 2020 statement: an updated guideline for reporting systematic reviews. Syst rev. 2021;10(1):1-1.

While in this instance, we aim to keep the PRISMA-P checklist 2015 in the manuscript (*systematic review protocol*), the subsequent systematic review will be reported according to the recent PRISMA 2020 guidelines (Please see lines 116-117).

-the PRISMA 2020 checklist should be followed in the exact same order, same sections and topics (as an example, the assessment of methodological quality should be removed. Instead, the Study risk of bias assessment should be added).
Thank you

Response: Thanks for raising this point. Since there is no significant difference in headings between the 2015 and 2020 checklists, the following section headings have now been renamed/updated (please see the tracked changes). The old sub-heading ‘assessment of heterogeneity’ has now been merged into ‘synthesis methods’ as per the order and headings of the PRISMA 2020 checklist:

- Information sources (line 153)
- Selection process (line 176)
- Data items (line 187)
- Study risk of bias assessment (line 206)
- Synthesis methods (line 227)
- Certainty assessment (line 244)

Please note that the section “Eligibility criteria” (line 119) has been moved up following the checklist order.

**Review Comments to the Author:**

Reviewer #1: The authors did a good job of reviewing the manuscript and answering all the revisions made.

Response: Thank you.

However, I think the paper could be more enriched if the authors could mention (briefly) how HRV can be measured in this type of population (civilian and military). We can use the same procedures?

Response: We appreciate your feedback. However, we would like to point out that this will be a systematic review and not a primary study. We do not aim to measure HRV in participants but present the results of previously reported work. However, we have cited references that provide a more in-depth review of HRV measurements and their interpretation. A detailed discussion on HRV measurement methods may be beyond the scope of this protocol. Nonetheless, we aim to address this in greater detail in the subsequent systematic review where it would be more relevant in the context of results.

Reviewer #2: I examined the Response "the Reviewer file". All corrections are made by the authors. Thank you so much for their effort. My decision is "accept"

Response: Thank you!
