# Supplementary material for: Association between non-acute traumatic injury (TI) and heart rate variability (HRV) in adults: A systematic review protocol
Source: PLoS One. 2022 Aug 26;17(8):e0273688. doi: 10.1371/journal.pone.0273688 (PMC9417186; doi:10.1371/journal.pone.0273688)
Supplement: Attachment — Submitted filename: Response to Reviewers.docx [file pone.0273688.s007.docx]

**PONE-D-22-12255R2:** **Association between non-acute traumatic injury (TI) and heart rate variability (HRV) in adults: a systematic review protocol**

We would like to thank the academic editor and the reviewers for their feedback. The suggestions made by the academic editor have been incorporated into the manuscript and are highlighted.

Please see below, in blue, for a point-by-point response to the reviewers’ comments.

ACADEMIC EDITOR:

Dear authors,
Congratulations! Both reviewers already accept the work in the current form while I still have a minor revision to suggest.

Response: Thank you!

Considering that this is a protocol for a systematic review that follows PRISMA-P 2015 (Moher, D., Shamseer, L., Clarke, M., Ghersi, D., Liberati, A., Petticrew, M., Shekelle, P., Stewart, L. A., & PRISMA-P Group (2015). Preferred reporting items for systematic review and meta-analysis protocols (PRISMA-P) 2015 statement. Systematic reviews, 4(1), 1. <https://doi.org/10.1186/2046-4053-4-1>) as you stated in the manuscript, I would like to suggest that authors also follow the same checklist item recommended in the reference.
For better clarity, after the section "Search strategy" you should present "Study records" in which you will include "data management" + "selection process" + "data collection process". After "Data items", it is suggested adding "Outcomes and
prioritization". "Study risk of bias assessment" should be replaced by "Risk of bias in individual studies". "Synthesis methods" should be replaced by "Data synthesis" as before. Then, you can add "Meta-bias(es)". Finally, instead of "Certainty assessment" used in the PRISMA 2020, it is suggest to replace by "Confidence in cumulative evidence".
I hope the authors can understand my suggestions.
After this step and if the authors follow these suggestions.
Thank you

Response: Thanks for raising this point. The revised manuscript now contains the suggested headings according to the PRISMA-P 2015 checklist. The following headings have been updated; line numbers are given in brackets.

Study records (174), Data management (175), Data collection process (190), Outcomes and prioritization (210), Risk of bias in individual studies (215), Data synthesis (233), Meta-bias (250), Confidence in cumulative evidence (254).

Please note a few changes made in the manuscript as a result of change in the headings:

- Lines 176-179 moved into the section of Data management
- Section on Outcomes and Prioritization added.
- Lines 191-202 moved into Data Collection Process
- Lines 251-253 moved into Meta-bias
- Reference numbers updated in the reference list and the text [35-39]
